# Supplementary material for: Dye-Encapsulated Metal–Organic Frameworks for the Multi-Parameter Detection of Temperature
Source: Molecules. 2023 Jan 11;28(2):729. doi: 10.3390/molecules28020729 (PMC9861431; doi:10.3390/molecules28020729)
Supplement: Supplementary file 1 [file molecules-28-00729-s001.zip › molecules-2018542-supplementary.pdf]

Electronic Supplementary Information for:

Dye Encapsulated Metal—Organic Frameworks for Multi-parameters Detection of Temperature

Yating Wan*, Yanping Li, Dan Yue*

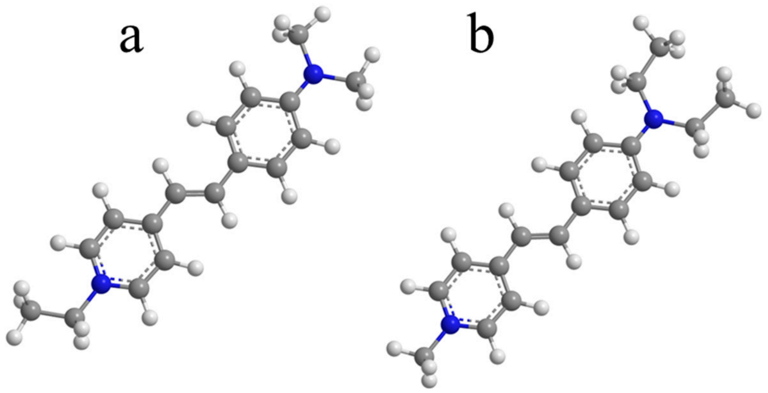

Figure S1. Structure of (a) DPEE, and (b) DPEM.

Table S1. Crystallographic Data collection and Refinement result for Zn-CYMPN.

Compound	Zn-CYMPN
chemical formula	$C_{98}H_{64}O_{24}Zn_3$
formula weight	1757.09
temperature (K)	293
radiation (Å)	0.71073
crystal system	Monoclonic
space group	I 2
a(Å)	21.2299(11)
b(Å)	26.9625(13)
c(Å)	21.6121(17)
α(°)	90
β(°)	97.432(5)
γ(°)	90

V(Å^3)	12267.1
Z	4
ρ(calc) (g/cm^3)	0.95134
F (000)	3480
absorp.coeff. (mm^{-1})	0.635
θ range (deg)	1.214 to 25.009
reflns collected	30513
indep. reflns	15770 [R(int) = 0.0627)
data/restr/paras	15770/1/1127
GOF	0.671
R1/wR2 [I＞2σ(I)]	0.0547/0.1264
R1/wR2 (all data)	0.1299/0.1527
largest peak and hole(e/Å^3)	0.223 and -0.282

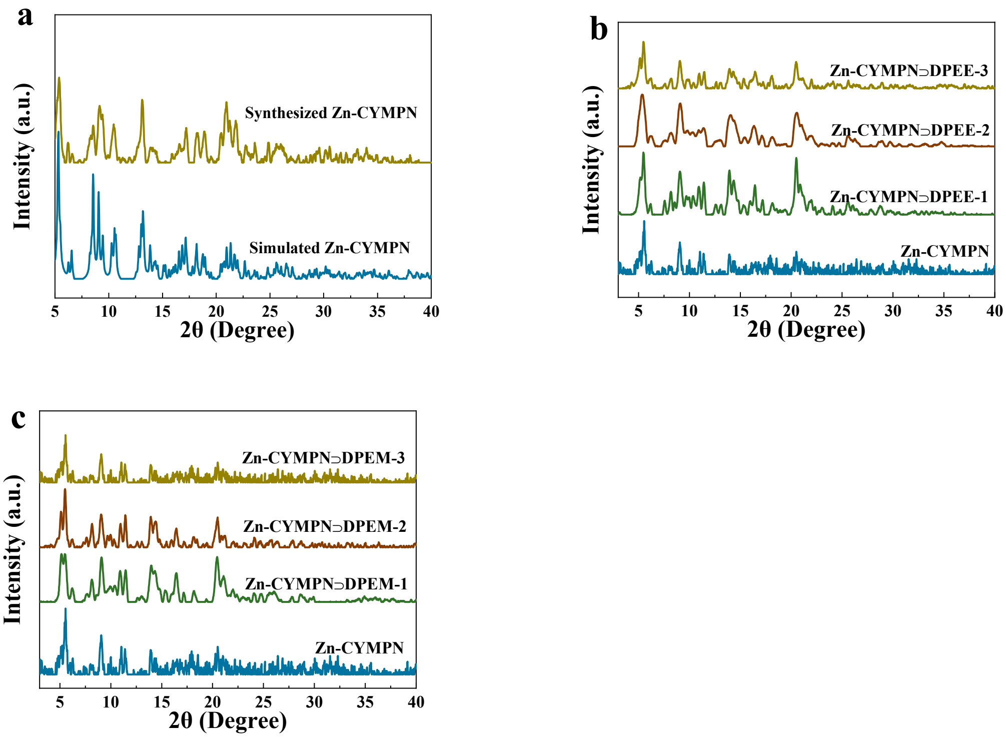

Figure S2. PXRD patterns of (a) simulated Zn-CYMPN and Zn-CYMPN as synthesized, (b) Zn-CYMPN as synthesized, **Zn-CYMPN⊃DPEE-1**, **Zn-CYMPN⊃DPEE-2**, and **Zn-CYMPN⊃DPEE-3**, (c) Zn-CYMPN as synthesized, **Zn-CYMPN⊃DPEM-1, Zn-CYMPN⊃DPEM-2**, and **Zn-CYMPN⊃DPEM-3**.

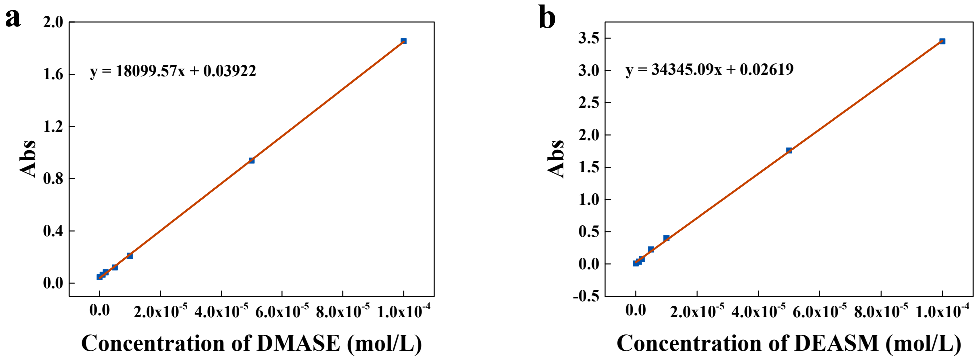

Figure S3. The intensity-concentration diagram and the fitting curves for (a) DPEE, (b) DPEM.

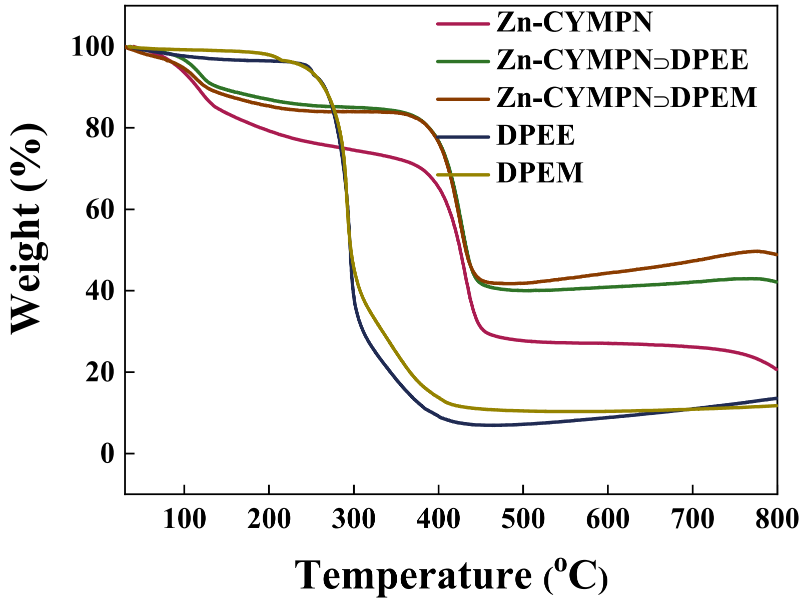

Figure S4. TG curves of Zn-CYMPN, **Zn-CYMPN⊃DPEE**, **Zn-CYMPN⊃DPEM**, DPEE and DPEM.

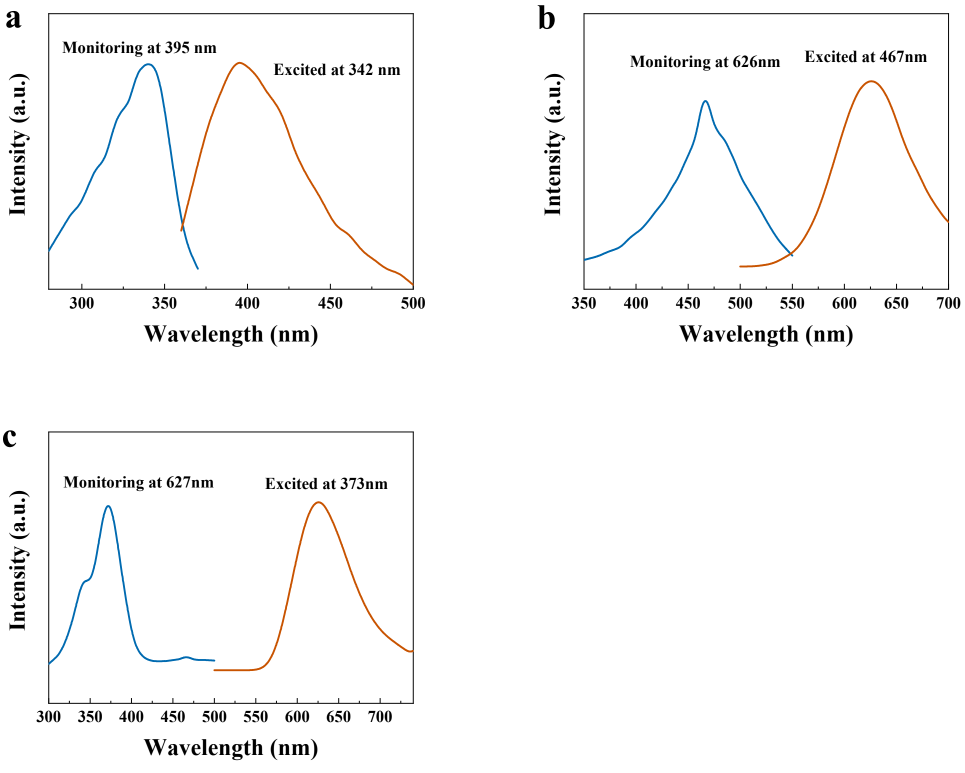

Figure S5. Excitation and emission spectra of (a) Zn-CYMPN, (b) DPEE, (c) DPEM.

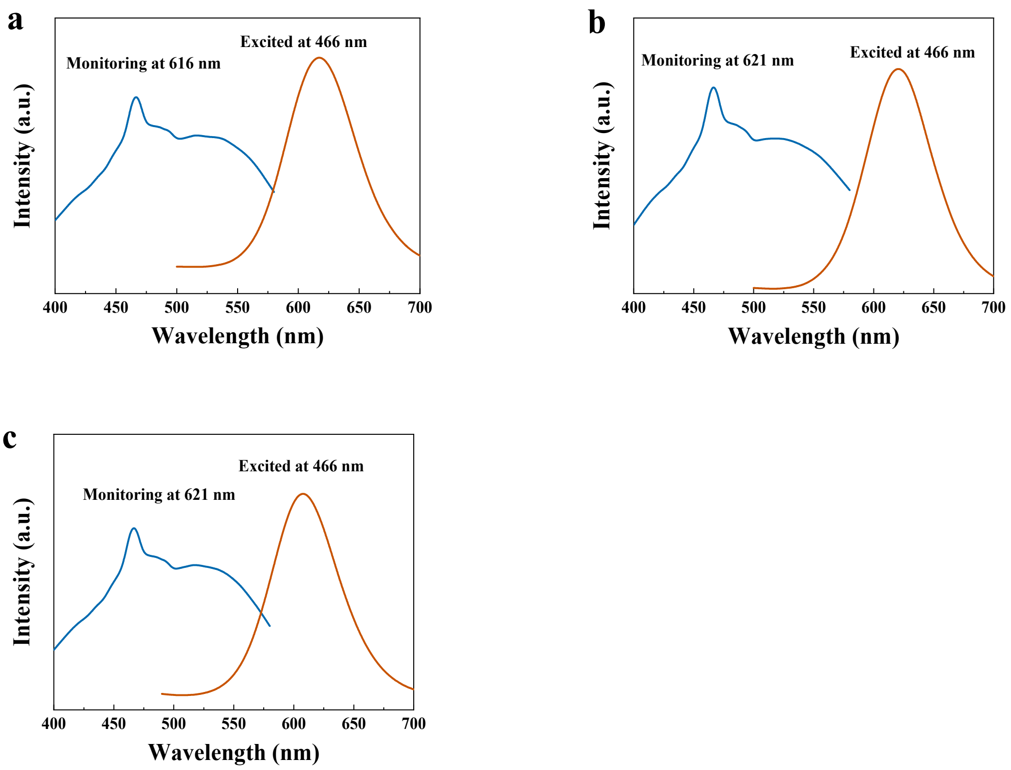

Figure S6. Excitation and emission spectra of (a) **Zn-CYMPN⊃DPEE-1**, (b) **Zn-CYMPN⊃DPEE-2**, (c) **Zn-CYMPN⊃DPEE-3**.

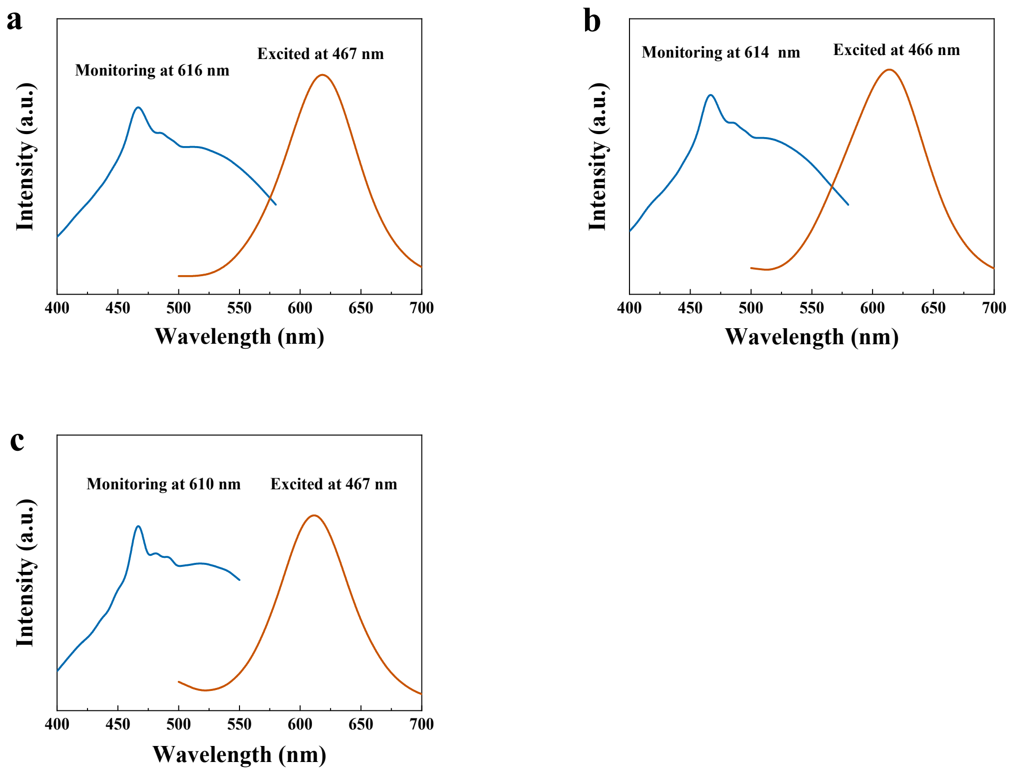

Figure S7. Excitation and emission spectra of (a) **Zn-CYMPN⊃DPEM-1**, (b) **Zn-CYMPN⊃DPEM-2**, (c) **Zn-CYMPN⊃DPEM-3**.

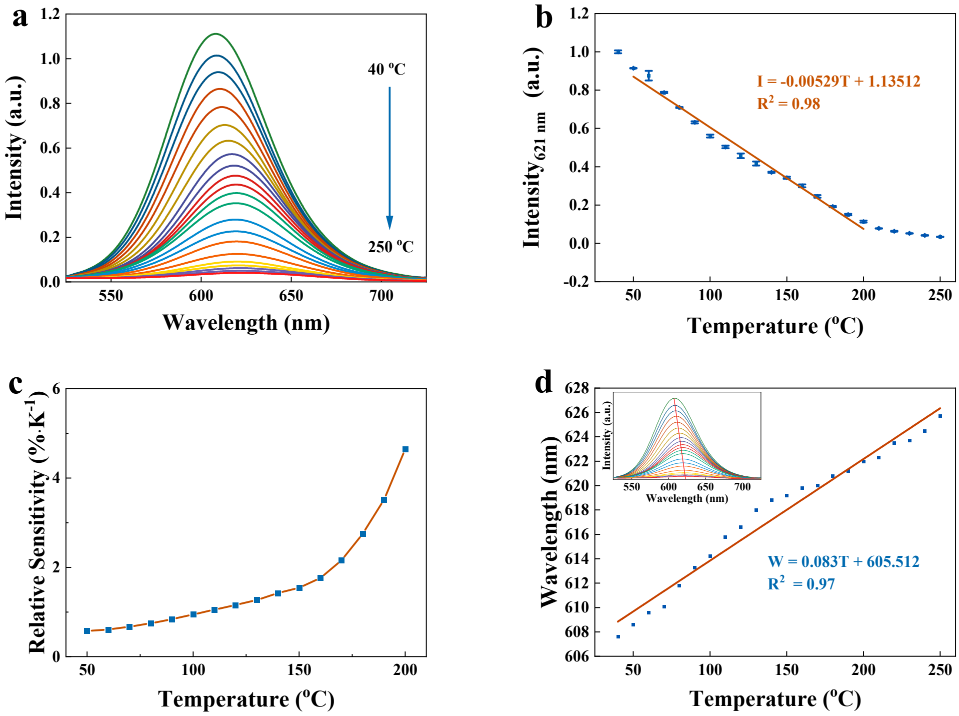

Figure S8. (a) Temperature dependent fluorescent emission spectra of **Zn-CYMPN⊃DPEE-2**. (b) Fluorescent intensity at 621 nm and the fitting line with temperature of 50~200 °C. (c) The relative sensitivity for intensity. (d) Wavelength at the maximum fluorescent emission and the fitting line with temperature of 40~250 °C (insert: temperature dependent fluorescent emission spectra and the shift of wavelength corresponding to the maximum fluorescence emission intensity).

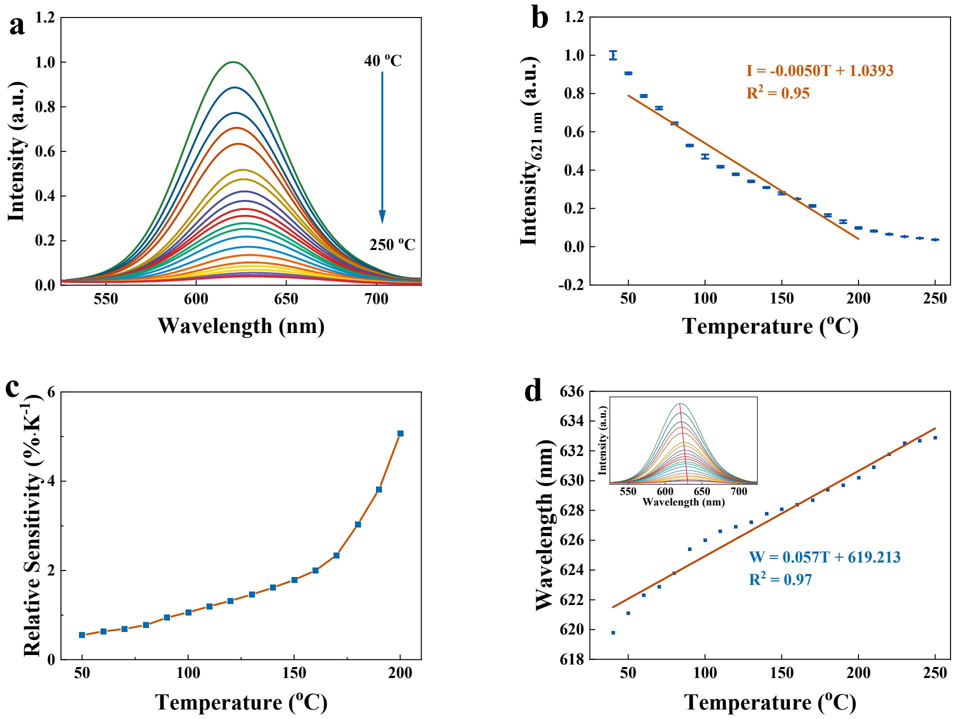

Figure S9. (a) Temperature dependent fluorescent emission spectra of **Zn-CYMPN⊃DPEE-3**. (b) Fluorescent intensity at 621 nm and the fitting line with temperature of 50~200 °C. (c) The relative sensitivity for intensity. (d) Wavelength at the maximum fluorescent emission and the fitting line with temperature of 40~250 °C (insert: temperature dependent fluorescent emission spectra and the shift of wavelength corresponding to the maximum fluorescence emission intensity).

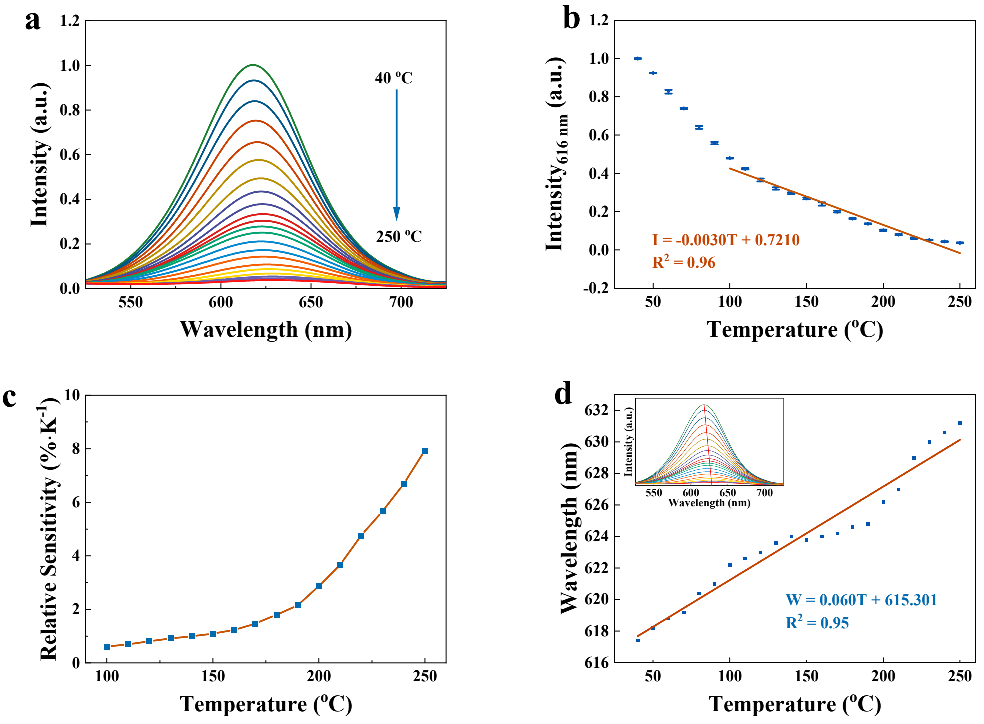

Figure S10. (a) Temperature dependent fluorescent emission spectra of **Zn-CYMPN⊃DPEM-1**. (b) Fluorescent intensity at 616 nm and the fitting line with temperature of 100~250 °C. (c) The relative sensitivity for intensity. (d) Wavelength at the maximum fluorescent emission and the fitting line with temperature of 40~250 °C (insert: temperature dependent fluorescent emission spectra and the shift of wavelength corresponding to the maximum fluorescence emission intensity).

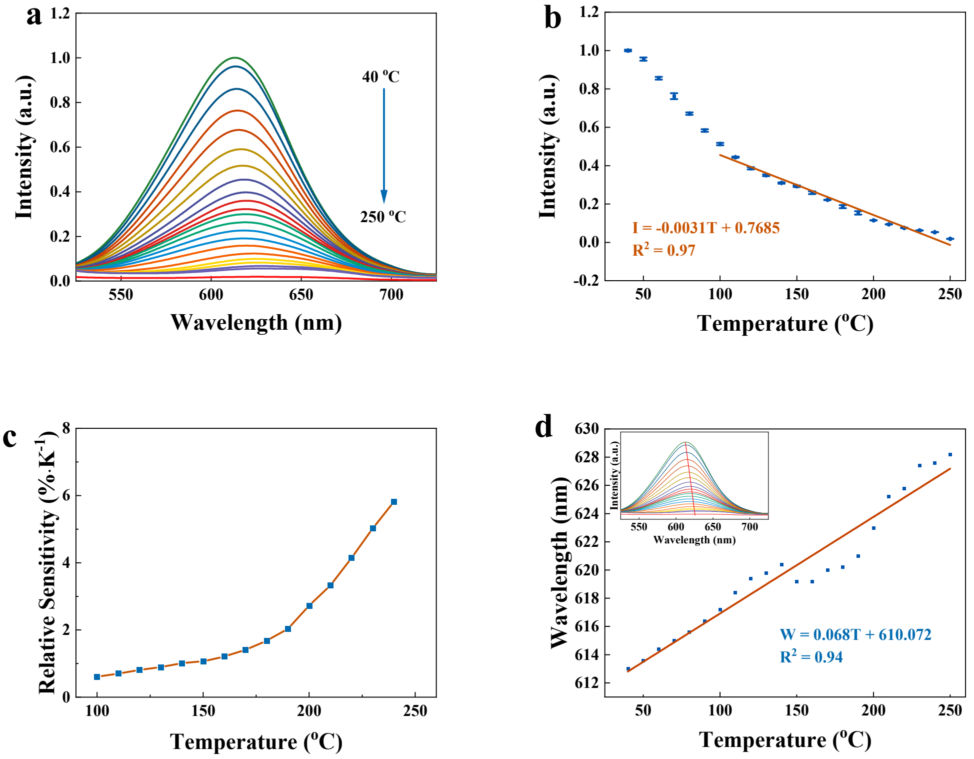

Figure S11. (a) Temperature dependent fluorescent emission spectra of **Zn-CYMPN⊃DPEM-2**. (b) Fluorescent intensity at 614 nm and the fitting line with temperature of 100~250 °C. (c) The relative sensitivity for intensity. (d) Wavelength at the maximum fluorescent emission and the fitting line with temperature of 40~250 °C (insert: temperature dependent fluorescent emission spectra and the shift of wavelength corresponding to the maximum fluorescence emission intensity).

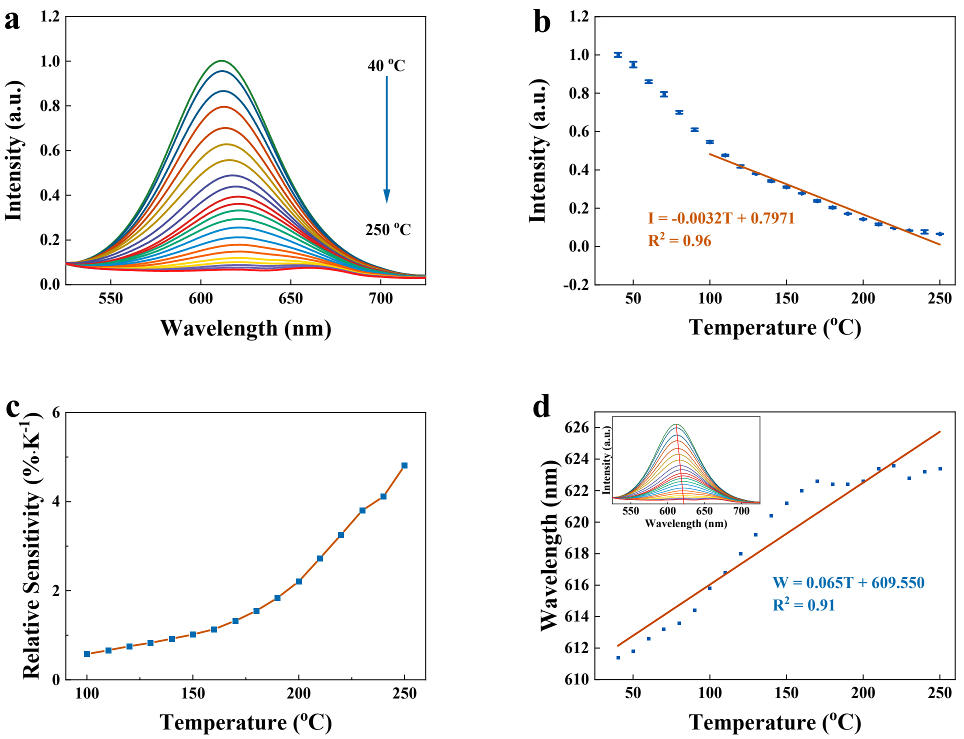

Figure S12. (a) Temperature dependent fluorescent emission spectra of **Zn-CYMPN⊃DPEM-3**. (b) Fluorescent intensity at 610 nm and the fitting line with temperature of 100~250 °C. (c) The relative sensitivity for intensity. (d) Wavelength at the maximum fluorescent emission and the fitting line with temperature of 40~250 °C (insert: temperature dependent fluorescent emission spectra and the shift of wavelength corresponding to the maximum fluorescence emission intensity).

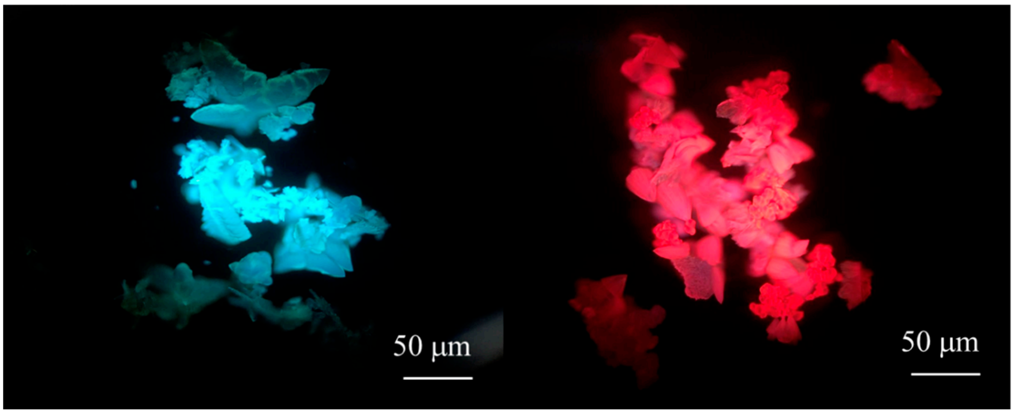

Figure S13. The optical photographs of Zn-CYMPN before (left) and after (right) encapsulated the **DPEE** with excitation at 380 nm mercury lamp.
